# Planning a sustainable neurosurgery mentorship program in a war-torn country: experience from Iraq

**DOI:** 10.1186/s41016-024-00376-1

**Published:** 2024-08-02

**Authors:** Teeba A. Al-Ageely, Mustafa Ismail, Zinah A. Alaraji, Jaafar Abdulwahid, Fatima Ayad, Huda Jaafar, Awfa Aktham, Hayder R. Salih, Samer Hoz

**Affiliations:** 1 College of Medicine, University of Baghdad, Baghdad, Iraq; 2 Department of Neurosurgery, Neurosurgery Teaching Hospital, Baghdad, Iraq; 3 College of Medicine, Al-Nahrain University, Baghdad, Iraq; 4 Department of Neurosurgery, Tokyo General Hospital, Tokyo, Japan; 5 Department of Neurosurgery, University of Pittsburgh Medical Center (UPMC), Pittsburgh, PA USA

**Keywords:** Medical education, Mentorship, War-torn countries, Neurosurgery

## Abstract

The importance of mentorships in medical education and neurosurgery is highly attributed to the support and encouragement of the advances and learning opportunities for medical students and junior neurosurgeons. Planning a mentorship program according to the target audience offers to satisfy different interests and enhance education. One of the main issues with most of the already implemented programs is the sustainability and inability to maintain continuous cycles of mentorship, which have a negative impact and have led to an interrupted pattern of learning which eventually leads to a decline in the engagement of participants and loss of interest. This problem is most pronounced in war-torn countries, with Iraq as an example, where external circumstances lead to an arrest in the educational process and a depletion of the resources useful for such programs and training courses. This paper aims to address the main pathways essential in planning a sustainable mentorship program in a war-torn country by highlighting our experience in maintaining an ongoing mentorship with nine consecutive courses over the last 6 years in Iraq.

## Background

Mentorships in medical education have been applied in multiple pathways to ensure support to medical students and junior doctors. A well-supported sustainable mentorship program is an efficient method to create an educational environment and encourage progress, especially in broad-ranged and delicate specialties like neurosurgery. In addition, the main objectives are to offer guidance and counseling to support the mentees and their future goals [1, 2]. Although such programs can be viewed as a natural and essential part of medical education, they are considered a luxury in areas of limited resources with a shortage of providers and mentors, particularly in war-torn countries like Iraq, where the availability and sustainability of such initiatives have become incredibly challenging. Moreover, planning and implementing mentorship programs may give opportunities to better support, motivate, and sustain the education and learning process flow, thus improving their academic achievements and providing orientation and a futuristic vision for students during the early stage of their career in medicine [3].

The first official neurosurgical training pathway in Iraq was relatively recently established in 1972, with the first tertiary neurosurgical center (Neurosurgery Teaching Hospital (NTH)) in the capital Baghdad. The hospital hosts 16 neurosurgeons (including 4 consultant neurosurgeons) and 20 neurosurgery residents, with an average of 2400 operations performed per year. Only a few other specialized centers have been established since then in Baghdad and a few other regions in Iraq. Furthermore, attempts at the development of this specialty in Iraq are mainly individual efforts with no national initiatives for further improvement [4, 5]. Considering the mentioned aspects above, educational programs in general and mentorship programs specifically directed toward neurosurgery are very limited and have a short-term continuity for multiple reasons like inadequate participation numbers, the lack of interest, and insufficient planning of such programs.

On the other hand, many implemented mentorship programs face difficulty maintaining sustainability through multiple cycles, especially in neurosurgery. This is significantly noted in war-torn countries with obstacles and distractive events that hone around such activities. This paper highlights the main pathways and steps that are prerequisites for planning a sustainable mentorship in a war-torn country from our experience with nine consecutive neurosurgery mentorship courses in Iraq over the last 6 years.

### Parameters attributing to the improvement of the quality of mentorship programs

There is a wide range of implications for mentorships in medical education. A few of those include improving the performance in medical schools, focusing on young medical students, and encouraging the development of female academics and minorities, as well as in-depth learning programs, attention to research interests, and early surgical exposure [3, 6, 7]. Planning for such programs will depend on the mentors’ experience and perspective on the mentorship goals. This can also vary according to the interests and academic levels of the applicants. Furthermore, it is widely influenced by the environment and availability of resources that might aid in learning progression and refinement of educational outcomes.

In our experience, the major pillars in creating a well-planned mentorship program include multiple parameters like the establishment of an integrated and flexible curriculum, the awareness of the participants’ fields of interest, involvement of students in course activities, the presence of motivational rewards to encourage passion and activity during the course, and the availability of interactive discussion panels to help participants express their opinion and feedback in every activity. In addition to fitting the participants’ interests and goals, these steps can further improve the quality of the programs as they progress and meet the goals set initially for the mentorship. Those settings are critical for sustainability by enhancing the participants’ engagement in the mentorship and aid in formulating an enlarging pool of new students already eager for the next cycle or program.

### Sustainability of mentorship programs

The main issue associated with most extracurricular programs for medical education is the inconsistency, leading to an interrupted pattern of learning that might cause participants to lose focus and momentum and eventually become uninterested in the program itself. Another problem with sustainability might include the repetition of contents and materials for such programs. Medical education is undeniably a continuous process with no obvious endpoint. Therefore, consistency and renewability of programs such as mentorships result in related material, better-gained knowledge, and an incline in interests over time. The concerns on sustainability are most pronounced in countries with unusual circumstances such as wars and national struggles, in which resources are depleted and the educational processes arrested, distracted, or interrupted.

In our experience, the sustainability of a neurosurgery mentorship program conducted by the last author of this paper nine times over the past 6 years in a war-zone region like Iraq has produced a solidified concept of the importance of the continuity of education despite the unusual circumstances and limited resources. During the 9 cycles of our neurosurgery mentorship programs, we succeeded in recruiting a total number of 1116 participants, and the majority of this number were medical students interested in neurosurgery as a future career choice; moreover, the mentorship also included postgraduates and neurosurgery residents. A total of 90% of the recruited pool is from Iraq, with 10% from 17 other countries around the globe. The paucity of accessible resources provided innovative opportunities for finding new teaching methods and modified training patterns. Each cycle takes approximately 10–12 sessions.

This has given this program high credibility resulting in a consistent rise in the number of interested individuals each year. It has also set higher standards and expectations for this mentorship program for participants, program directors, and coordinators, which turn the program’s planning into a brainstorming experience and a process of “doing more with less” as it originated from Iraq, a country with minimal resources and funding such initiatives; this point, in particular, is what distinguishes this experience from training programs for young physicians taking place in more developed countries like the United States and China.

It is important to note that there were no constant or redundant schedules regarding topics discussed during the mentorship each cycle. This was intended to ensure innovation and the unique experience of each batch. The topics were tailored to meet the needs and peak the interests of the vast majority of students, residents, and specialists involved. Therefore, the topics would range from basic anatomical orientation to precise neurosurgical elements and operative views. Multiple clinically applied topics like cerebrovascular surgery, traumatic brain injury, neuro-oncology, neurophysiology, and critical care were all discussed. The program was conducted by the last author of this paper (H. S.), a senior neurosurgeon with many years of experience in the neurosurgical field, medical education, and research; therefore, a cornerstone of the program in every cycle is the clinical research and medical writing skills, which provided participants with enough knowledge to start contributing to many research papers.

Multiple neurosurgeons from all around the world and with different specialties were hosted through an online platform to contribute with presentations relaying their experience and life with neurosurgery as a career choice, and in-depth analysis of different neurosurgical subspecialties, as well as discussing many therapeutic options employed in neurosurgery. Some residents were also involved in presentations to highlight their own experiences and their views on residency and training in neurosurgery. Surgical training skills also had a major role in multiple cycles. The mentorship also included case discussions which had a major part in the educational process of this program because of the associated multitudes of clinical, theoretical, and ethical aspects which all serve as good teaching points regardless of the academic levels of the participants [8]. The chief goal of this mentorship program is to provide orientation to medical students and even residents regarding the field being considered as a future career path to make a more informed decision in the future. Although the neurosurgical knowledge gained is significant, it is important to note that participants still require adequate clinical training to reach the residency level.

### Potential pursued pathways when applying to mentorship programs

There are multiple potential pathways mentees seek in their application to mentorship programs. In our ninth conducted neurosurgery mentorship program, we found that most applicants’ main goal is the observation of the provided topics out of curiosity; most of this category is represented by early-stage medical students. This can be partially attributable to medical students primarily seeking medical education mentorship programs as an additive extracurricular opportunity to enhance their academic achievements. There are variable reasons why medical students apply to such mentorship programs, the main motive being curiosity and having the opportunity to learn more about variable specialties, although such opportunities are limited to medical students especially in Iraq, as knowledge about many specialties is only gained through the limited scope of medical school. It is also important to note that many participating students apply to programs related to other specialties of their interests when they have the chance.

Another common application reason includes the applicants’ interest in gaining a mentor’s guidance throughout their medical education and specialty orientation. Additional purposes include interest in the field of neurosurgery as a specialty and aiming for further knowledge and experience, the focus on building one’s resume by participating in activities provided by mentors such as research enrollment, and the interest in making connections; this is especially pronounced in mentorship programs with international standards.

## Methods

During the ninth cycle of the mentorship program, a survey was formulated for participants to fill out, and the survey included questions regarding the demographic data of the participants (age, nationality, affiliation, and levels of education) as well as neurosurgical knowledge and experience. Additionally, all the applicants attached a motivational letter illustrating the purpose of their application to such a program.

## Results

Upon analyzing the participants through their application letters and the given survey, we found that most of the applicants (82%) were from the local country where the mentorship initially originated, Iraq. This number highlights the developed concept of the importance of persistence in medical education, especially in war-torn countries, even in the mindsets of medical students. Moreover, approximately 18% were international applicants from 18 different countries. Regarding the levels and the age groups, the vast majority of the participants (85%) were medical students from varying stages, 7% were recent graduates from medical school, and the remaining 8% were higher-leveled participants (interns, residents, and general practitioners).

According to their motivational letters, some shared goals were found regarding the interests and goals of the applicants. A total of 46% of the participants were considered curious, with their primary interest in reflection and learning more about the basics of the specialty of neurosurgery. A total of 29% had a genuine interest in the specialty and sought to make an informed decision regarding their future career plans. A total of 12% of the participants were interested in the mentorship idea and considered the importance of having a mentor during their medical education, regardless of their interests and future specialty. A total of 9% of the participants were more focused on their resumes and were interested in building them through the opportunities offered by the mentorship program. The remaining 2% of applicants have goals focused on building connections and networking with international individuals with similar interests and plans.

This categorization can help understand the patterns of applications, the principal shared goals, the direction of attention, and the educational level presented during the sessions. It also helps to build more knowledge about the participants and implement plans that can assist them in achieving their goals. This is also beneficial in understanding the participants’ demographics for further attention to the language differences and time zones. Most activities were online due to the pandemic and continued after that.

Based on the above results, the focus of our mentorship program was directed toward medical students with the implication of different topics that can simultaneously satisfy the interest of higher-level interns, residents, and physicians. These planning methods can give a clear direction for the mentorship program and its target audience, as well as support the two-way relationship between the mentors and mentees by highlighting their interests, skills, and goals for mentors. This experience also builds the pillars for ongoing and future employed programs which might greatly benefit from expanding into partnering with other institutions regionally that can provide both neurosurgical orientation and surgical training skills improvement. International collaborations can also be of great value in conferences and implementing the importance of networking and more advanced training opportunities.

## Conclusion

Mentorship programs in medical education are essential in the early exposure of students and residents to their specialty of interest. This can be a challenge in countries with lower income or unusual circumstances like war zones where the sustainability of programs is significantly affected. Planning and analyzing the vision and the objectives of mentorships based mainly on the participants, and the mentors’ experiences significantly raise the reliance and sustainability of such projects.

## Data Availability

Not applicable as no datasets were generated or analyzed during the study.
